# A 35 nV/√Hz Analog Front-End Circuit with Adjustable Bandwidth and Gain in UMC 40 nm CMOS for Biopotential Signal Acquisition

**DOI:** 10.3390/s24247994

**Published:** 2024-12-14

**Authors:** Lu Liu, Bin Wang, Yiren Xu, Xiaokun Lin, Weitao Yang, Yinglong Ding

**Affiliations:** State Key Discipline Laboratory of Wide Bandgap Semiconductor Technology, School of Microelectronics, Xidian University, Xi’an 710071, China

**Keywords:** capacitively-coupled chopper instrumentation amplifier, low noise, dc servo loop, ripple reduction loop, bandwidth-gain adjustable, biopotential signal acquisition

## Abstract

This paper presents a 35 nV/√Hz analog front-end (AFE) circuitdesigned in the UMC 40 nm CMOS technology for the acquisition of biopotential signal. The proposed AFE consists of a capacitive-coupled instrumentation amplifier (CCIA) and a combination of a programmable gain amplifier (PGA) and a low-pass filter (LPF). The CCIA includes a DC servo loop (DSL) to eliminate electrode DC offset (EDO) and a ripple rejection loop (RRL) with self-zeroing technology to suppress high-frequency ripples caused by the chopper. The PGA-LPF is realized using switched-capacitor circuits, enabling adjustable gain and bandwidth. Implemented in theUMC 40 nm CMOS process, the AFE achieves an input impedance of 368 MΩ at 50 Hz, a common-mode rejection ratio (CMRR) of 111 dB, an equivalent input noise of 1.04 μVrms over the 0.5–1 kHz range, and a maximum elimination of 50 mV electrode DC offset voltage. It occupies an area of only 0.39 × 0.47 mm^2^ on the chip, with a power consumption of 8.96 μW.

## 1. Introduction

With the rapid development of integrated circuits and biomedical technology, biomedical sensor chips have become a popular research direction in the medical field, primarily applied to the real-time detection of biopotential signals such as ECG and EEG [1,2,3,4,5]. Bioelectric signals are low-frequency and low-amplitude electrical signals, mainly distributed in the frequency band from DC to a few kHz, with amplitudes ranging from tens of microvolts to tens of millivolts [6]. To effectively acquire these biopotential signals, the AFE often needs to meet the following characteristics: low noise, low power consumption, suppression of EDO, high input impedance, and a high CMRR [7].

**Figure 1:**
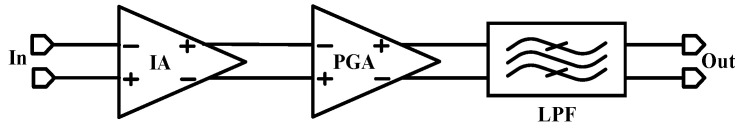
Block diagram of a typical biopotential signal acquisition system.

The typical architecture of an analog front-end for biopotential signal acquisition is shown in Figure 1, and mainly consists of three modules: an instrumentation amplifier (IA), a programmable gain amplifier (PGA), and a low-pass filter (LPF). The instrumentation amplifier is a key module that determines the performance of the analog front-end, and various related topologies have been proposed. The classical three-opamp topology can achieve high input impedance, high CMRR, and gain accuracy [8]. However, the three-opamp structure limits its power efficiency compared to other single-opamp solutions. For the current feedback instrumentation amplifier, the transconductance of the input transconductor can vary with a large input common mode (CM) range, thus reducing gain accuracy [9]. Recently, to eliminate the various trade-offs associated with resistive feedback, CCIA has been proposed to realize a wide input CM range and high gain accuracy [10]. Thus, the capacitively coupled structures are more suitable for designing analog front-ends for biopotential signal acquisition.

The bandwidth of the biopotential signals is typically within hundreds of hertz, and flicker noise becomes significant in this frequency range. Chopper modulation is the most common technique for suppressing the contribution of the flicker noise [11,12,13,14,15]. However, the capacitively coupled chopper instrumentation amplifier (CCIA) has some fundamental drawbacks: electrode DC offset voltage, chopping ripple, limited input impedance, etc. To suppress EDO, the DC servo loop (DSL) technology is often utilized [4,11]. The key issue in DSL is implementing an oversized time-constant integrator to create a sub-Hz high-pass turning frequency [16]. Conventional DSL often uses pseudo-resistive (PR) integrators and very large time-constant switched capacitance (VLTSC) integrators [3,17]. The uncontrollability of the PR resistor can result in a rather inaccurate high-pass turning frequency for CCIA. An accurate high-pass turning frequency for CCIA can be obtained based on the VLTSC integrator, but it increases the input reference noise (IRN) of CCIA from 0.7 to 6.7 μVrms over the range of 0.5–100 Hz, as described in [17].

The chopping technology cannot remove low-frequency noise and DC offsets; it only modulates these disturbances to a higher frequency [18], potentially resulting in a large ripple inthe output. Therefore, the removal or suppression of the IA output ripple is critical and is usually eliminated using a ripple rejection loop (RRL). In [19], an SAR-assisted ripple reduction technique is employed to reduce the ripple. This approach improves performance by a factor of 1.3 compared to state-of-the-art IAs, yet it results in a power consumption of 5.35 mW, which does not meet the requirements for low-power design. Additionally, the operation of the input chopper charges and discharges the input capacitor C_in_, generating large charge and discharge currents. This significantly reduces the input impedance (typically less than 100 MΩ) and seriously affects the acquisition of biological signals [14,16,20]. Lastly, the IA must have a high common-mode rejection ratio (CMRR) above 80 dB to counteract common-mode noise and 50/60 Hz power-line interference [19].

In this paper, we introduce a 35 nV/√Hz analog front-end (AFE) circuit designed for biopotential signal acquisition with adjustable bandwidth and gain. The AFE is composed of a CCIA and an LPFintegrated with a PGA. The capacitively coupled instrumentation amplifier utilizes the global chopping technology in which the input chopper is located before the input capacitance. The CCIA can significantly enhance the noise performance of the circuit and achieve a high CMRR. To further optimize circuit performance, we have integrated transconductance enhancement technology, a DC servo loop (DSL) employing duty-cycle resistors, and a self-zeroing ripple rejection loop (RRL) into the CCIA. The PGA-LPF module features a circuit combination design that further reduces the overall power consumption of the analog front-end. Additionally, each module within the AFE employs a fully differential structure to enhance the system’s CMRR.

This paper is organized as follows: Section 2 describes the overall architecture of the proposed AFE. Section 3 presents the CCIA design details and circuit analysis, and Section 4 gives the design principles and implementation of the PGA-LPF. The post-simulation results of the system circuits are given in Section 5. Finally, the study is summarized in Section 6.

## 2. Overall Architecture Design 

**Figure 2:**
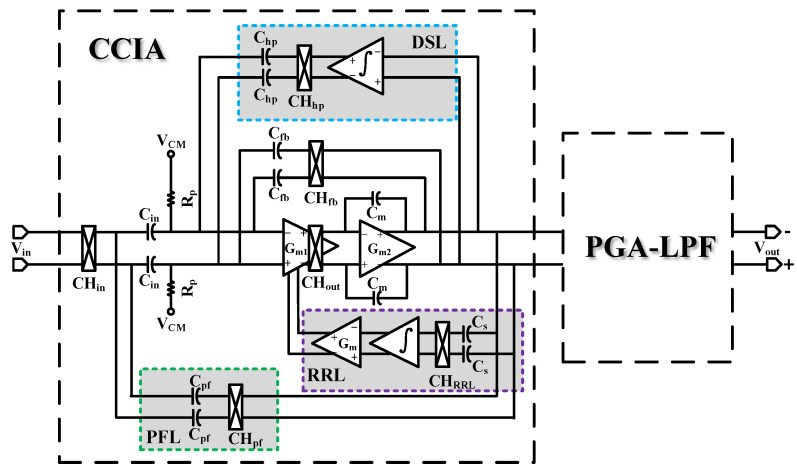
Proposed AFE for biopotential signal acquisition.

As shown in Figure 2, the proposed AC-coupled AFE circuit mainly consists of a capacitively coupled chopper instrumentation amplifier (CCIA), a capacitive feedback programmable amplifier (PGA), and a switched-capacitor low-pass filter (LPF);the PGA is integrated with the LPF in the design. 

For the CCIA, *C_in_* and *C_fb_* are the input and feedback capacitors of the main amplifier, respectively. Therefore, the mid-band gain Amid-band can be expressed as

(1) $$A_{CCIA}=\frac{A_{0}}{1+A_{0}\frac{C_{fb}}{C_{in}}}\approx \frac{C_{in}}{C_{fb}}$$

where *A*_0_ is the open-loop gain of the operational amplifier. In this paper, an input capacitance *C_in_* of 12 pF and a feedback capacitance *C_fb_* of 120 fF are chosen to achieve a closed-loop gain of 40 db. In addition, the Miller compensation capacitor C_m_ is designed to ensure the robustness of the op-amp closed loop. The chopper modulation technique suppresses the input DC offset and 1/f noise of the op-amp. 

Due to the DC coupling characteristics, the input impedance of the AFE amplifier should also be reduced to 1/(2f_chop_*C_in_), where *f_chop_* is the chopping frequency with a value of 5 kHz. With an input capacitance size of 12 pF, the equivalent input impedance of the instrumentation amplifier is about 8.3 MΩ, which is not up to the specification requirements for biological signal acquisition [5]. As shown in Figure 2, to improve the input impedance, a classical positive feedback loop (PFL) is used in this design [19]. When C_pf_ = 121.12 fF, the equivalent input impedance can reach infinity. However, it is difficult to achieve such an accurate capacitance in a real circuit. Thus, we choose *C_pf_* = *C_fb_* = 120 fF, which can increase the equivalent input impedance by a factor of 100.

To obtain lower power consumption, a subthreshold design is used for the input transistors of the main amplifier of the CCIA. For the problem of DC isolation failure of the global chopper amplifier circuit, a DC servo loop is introduced to suppress the electrode DC offset. Since the output high-frequency ripple generated by the chopper may cause the original signal to be overwritten, a ripple rejection loop (RRL) is introduced to suppress the output ripple. The PGA-LPF module provides 0~12 dB gain and 250~1 kHz bandwidth adjustability, and the AFE gain and frequency are adjusted to amplify biological signals of different frequencies to the appropriate amplitude range. The PGA-LPF module realizes low-pass filtering and adjustable gain amplification of signals with only one circuit module, which effectively reduces the circuit area as well as power consumption. It also employs gate bootstrap switching to obtain better linearity and accuracy. Detailed circuit design and analysis will be discussed later in the subsequent sections.

## 3. Analysis and Design of the Proposed CCIA

### 3.1. Design of the Main Op-Amp

**Figure 3:**
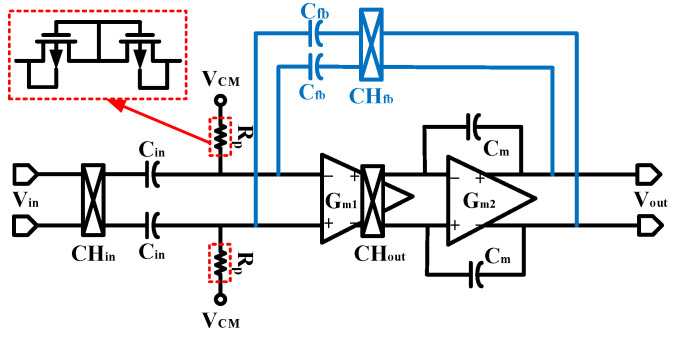
The schematic of the employed instrumentation amplifier.

The main amplification unit of the instrumentation amplifier, which utilizes a capacitive-coupled structure with global chopping, is shown in Figure 3. It consists of the main operational amplifiers (G_m1_ and G_m2_), the capacitive feedback networks (C_in_ and C_fb_), the input chopper switch (CH_in_), the feedback chopper switch (CH_fb_), the internal chopper switch of the operational amplifier (CH_out_), and the pseudo-resistor (RP) to provide DC bias. 

The capacitively coupled structure circuit requires a resistor with a resistance value of up to gigaohms (GΩ) to provide DC bias. Such a large resistor cannot be integrated on-chip, so a MOS-Bipolar structure pseudo-resistor is usually used to act as a large resistor to provide DC bias [11]. The pseudo-resistor consists of two PMOS transistors connected to a diode, which can achieve impedances of up to GΩ over a small area. When the voltage difference across the pseudo resistor is very small, the two MOS transistors operate in the deep subthreshold region. At this point, the transistor is turned off and no current is flowing, so it can be equated to a large resistance. The value of its resistance is mainly determined by the leakage current of the transistor. Due to the pseudo-resistor resistance value of up to GΩ, the gate leakage current will cause a large voltage difference across it. In the UMC 40 nm CMOS process, the gate leakage current of the thin gate transistor is usually a few hundred fA, and the maximum can reach several pA. For example, a pseudo-resistor impedance of 100 GΩ will produce a voltage difference of up to 100 mV. This would be fatal to the effects of bio-signal amplification. However, the gate leakage current of thick gate transistors is reduced by at least three orders of magnitude compared to thin gate transistors. Therefore, to avoid significant bias voltage errors, the input pairs of op-amps in this paper use thick-gate transistors. Although the threshold voltage of thick-gate transistors is larger, and the circuit design is more difficult at low supply voltages, the effect of using a pseudo-resistor to provide bias gate leakage currents at this time can be ignored.

**Figure 4:**
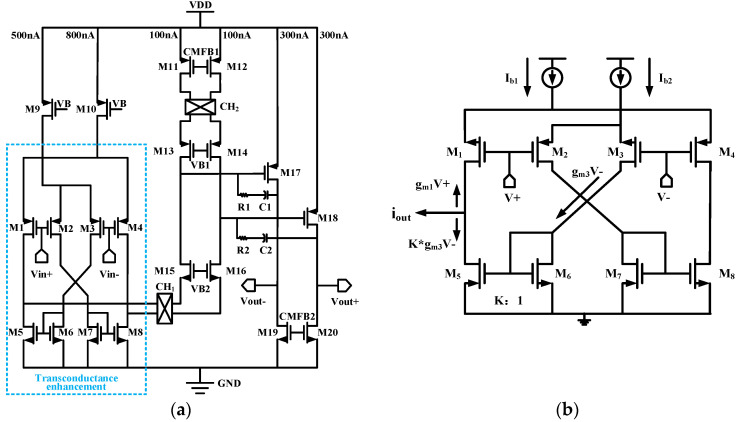
(**a**) Schematic of employed instrumentation amplifier. (**b**) Diagram of transconductance enhancement technology.

To obtain a gain accuracy of at least 0.1%, the open-loop gain of the amplifier needs to be higher than 100 dB; therefore, G_m1_ selects a two-stage Miller-compensated operational amplifier, as shown in Figure 4a. The op-amp adopts a two-stage amplifier structure, with the first stage using a folded cascode structure to provide a higher gain. The second stage uses a common-source stage to provide an additional gain as well as a large output swing. Also, G_m1_ contains two chopper switches, CH_1_ and CH_2_; CH_1_ is located at the source of M15 and M18, and CH_2_ is located at the drain of M11 and M12.The input stage of G_m1_ employs a transconductance-boosting technique to increase the equivalent transconductance of the circuit [3].

The principle of the transconductance enhancement technique is shown in Figure 4b. Analyzed with a half-side circuit, the input signal *V+* is converted to an output current through M1, with the magnitude of the current being *g_m_*_1_*V+*. Meanwhile, the reversed input signal *V-* is converted to a current through M3, with the magnitude of the current being gm3V-, and the current then passes through M6 and replicates K times to M5. Thus, the equivalent input transconductance is as shown below:

(2) $$g_{m}^{\prime }=g_{m1}+Kg_{m3}$$

where *K* represents the aspect ratio of transistors M5 and M6. In this paper, we chose *K* = 2.

For the transimpedance amplifier G_m1_, the chopper switch CH_in_ demodulates the input signal modulated to high frequencies back to the fundamental frequency. At the same time, the noise and mismatch of the input pairs of transistors M1~M4 and current mirrors M5~M8 are modulated to high frequencies. The chopper switch CH_out_ modulates the 1/f noise and mismatch of the current mirror M11~M12 to high frequencies. The noise of the cascode transistors M13~M16 is usually negligible. Most of the 1/f noise and G_m1_ distortion are modulated to high frequencies and separated from the signal. For the transconductance amplifier G_m2_, the noise equivalent of G_m2_ is divided by the gain of G_m1_ at the output of the op-amp, thus the noise of G_m2_ is also negligible. In addition, the op-amp input uses a PMOS transistor input, which has better 1/f noise performance than NMOS transistors. 

Considering the thermal noise of G_m1_ and analyzing the one-sided circuit noise of G_m1_, the magnitude of the thermal noise of the input transistor M1 is

(3) $$\overset{-2}{v}n,M1=\frac{4kT\gamma }{gm1}$$

where *k* is the Boltzmann constant, *T* is the Kelvin temperature, g_m_ is the transconductance of the MOS transistor, and *γ* is the process factor. 

The equivalent input noise magnitudes for M3 and M6, ignoring 1/f noise, is

(4) $${\overset{-2}{v}}_{n,M3/6}=K^{2}\frac{4kT\gamma gm3,6}{g_{m1}^{2}}$$

where *K* denotes the ratio of M6 to M5 width to length ratio.

(5) $$K=\frac{\left(W/L\right)M6}{\left(W/L\right)M5}$$

The equivalent input noise magnitude of M5 and M11 is

(6) $${\overset{-2}{v}}_{n,M5/11}=\frac{4kT\gamma g_{m5,11}}{g_{m1}^{2}}$$

Because the noise of cascode transistors M13 and M15 is negligible, the equivalent input noise of G_m1_ can be expressed as follows:

(7) $${\overset{-2}{v}}_{n,Gm1}=8kT\gamma \left(\frac{1}{g_{m1}}+K^{2}\frac{gm3+gm6}{g_{m1}^{2}}+\frac{gm5+gm11}{g_{m1}^{2}}\right)$$

From the above equation, it can be seen that reducing the equivalent input noise of the transconductance amplifier G_m1_ can be achieved by increasing the input transconductance gm1 by increasing the input stage current while reducing the currents of the load transistors M_5_ and M_11_ to reduce their transconductance values. In the case of the current-mirror transistors M_3_ and M_6_, since their currents are limited by the ratio of the mirror currents K as well as by the input stage currents, it is possible to obtain a relatively low transconductance value by decreasing their width-to-length ratios.

### 3.2. Design of DC Servo Loop (DSL)

The excessive DC out-of-phase will cause the instrumentation amplifier to fail to operate properly [4,11,12]. The CCIA circuit structure uses a global chopper, and the chopper switch is in front of the input capacitor. The clock switching in the chopper will cause the input capacitor to be charged and discharged, which will lead to a failure of the instrumentation amplifier’s isolation function. 

**Figure 5:**
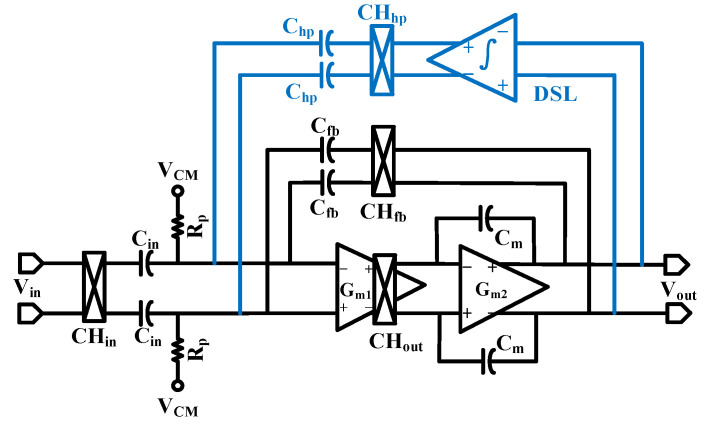
The structure of the instrumentation amplifier with a DC servo loop.

The circuit structure of the instrumentation amplifier with DSL is shown in Figure 5 andmainly consists of an integrator (int), a chopper switch (CH_hp_), and a capacitor (C_hp_). The EDOvoltage at the input of the instrumentation amplifier is superimposed on the output in a DC state by the chopper switch. The integrator maintains the integration of the DC voltage at the output, and the integrated voltage is modulated to a high frequency by the chopper switch CH_hp_. It is converted to a current by the capacitor C_hp_ to offset the current generated by the DC distortion voltage, and the integrator continues to integrate until there is no DC at the output of the instrumentation amplifier. 

When the current fed back from the DSL to the input of the op-amp is canceled by the current generated by the DC offset voltage, it gives the following equation:

(8) $$V_{EOV}=\frac{C_{hp}}{C_{in}}V_{\mathrm{int},\max}$$

where *V*_EOV_ is the maximum electrode DC offset voltage that can be eliminated by the DSL and *V_int,max_* is the maximum output voltage of the integrator. In order to eliminate the 50 mV electrode offset voltage at a supply voltage of 1.2 V, the *C_hp_* must be at least 416 fF.

As shown in Figure 5, the DSL is introduced in the instrumentation amplifier, and the following equation can be derived from *KCL* at the input of the main op-amp:

(9) $$V_{in}sC_{in}+V_{out}sC_{fb}+V_{out}\frac{1}{sR_{\mathrm{int}}C_{\mathrm{int}}}sC_{hp}=0$$

Thus, the closed-loop transfer function added to the instrumentation amplifier is

(10) $$V_{out}=-V_{in}\frac{C_{in}}{C_{fb}}\frac{sR_{\mathrm{int}}C_{\mathrm{int}}\frac{C_{fb}}{C_{hp}}}{1+sR_{\mathrm{int}}C_{\mathrm{int}}\frac{C_{fb}}{C_{hp}}}$$

Equation(10) shows that the DSL introduces a high-pass pole in the instrumentation amplifier, and the high-pass cutoff frequency is

(11) $$f_{hp}=\frac{1}{2\pi R_{\mathrm{int}}C_{\mathrm{int}}}\frac{C_{hp}}{C_{fb}}$$

According to Equation(11), the high-pass cutoff frequency of the instrumentation amplifier is determined by the unit gain bandwidth of the integrator and by the ratio of *C_hp_* and *C_fb_*, whereas the value of *C_fb_* cannot be readily changed due to the determination of the closed-loop gain. The value of *C_hp_* influences the maximum magnitude of the electrode DC offset that can be suppressed by the DSL and is usually also a fixed value. Thus, the high-pass poles affecting the instrumentation amplifier are primarily determined by the unit gain bandwidth of the integrator. 

To avoid the influence of the high-pass pole on the effective biological signal, it is usually necessary to ensure that the high-pass pole is less than 0.5 Hz. According to Equation (11), it can be seen that the unit gain frequency of the integrator is at least 0.1 Hz.

**Figure 6:**
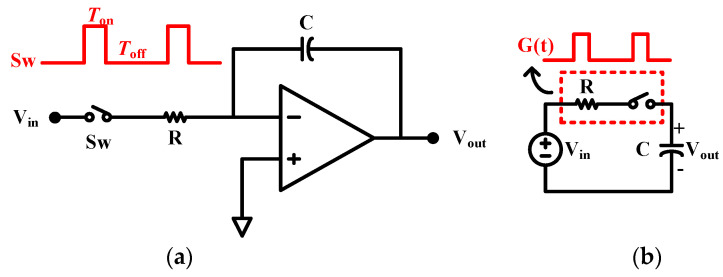
Utilized duty cycle resistor of (**a**) integrator and (**b**) low-pass filter.

Therefore, the integrator of the DSL in this paper uses a duty cycle resistor to achieve very large time constants in a small area, and its structure, which consists of a resistor R and a switch S_w_, is shown in Figure 6a.

(12) $$D=\frac{T_{on}}{T_{on}+T_{off}}$$

where *D* is the duty cycle factor of the clock controlling the switch, *T_on_* is the switch opening time during the cycle, and *T_off_* is the switch-off time during the cycle; thus, the equivalent resistor *R_eq_ = R/D*. Since the duty cycle resistor is time-varying, frequency shift and aliasing effects need to be taken into account. They can be analyzed based on the low-pass filter constructed from the duty cycle resistor in the species of Figure 6b. Assuming that *G (t)* is the time-varying conductance of the duty cycle resistor, the following equation is obtained using KVL:

(13) $$V_{in}(t)\cdot G(t)=V_{out}(t)\cdot G(t)+C\frac{dV_{out}}{dt}$$

Analyzed in the frequency domain perspective, the Fourier transform of *G(t)* is given by the following equation:

(14) $$G(j\omega )=\sum_{k=-\infty }^{\infty }g_{k}\delta (\omega -k\omega_{0})$$

In Equation (14), g_0_ = *D/R* and g_k_ is the Fourier coefficient corresponding to the waveform *G(t)* with the following expression:

(15) $$g_{k}=\frac{1}{\left|k\right|\cdot R\cdot \pi }\cdot \sin\left(D\cdot k\cdot \pi \right)$$

The Fourier transform of Equation (13) yields the following equation:

(16) $$g_{0}V_{in}(j\omega )+\sum_{\begin{array}{l} k=-\infty \\ k\neq 0 \end{array}}^{\infty }g_{k}V_{in}(j(\omega -k\omega_{0}))=(g_{0}+j\omega C)V_{out}(j\omega )+\sum_{\begin{array}{l} k=-\infty \\ k\neq 0 \end{array}}^{\infty }g_{k}V_{out}(j(\omega -k\omega_{0}))$$

To simplify the analysis, assuming that *RC* ≫ *DT*, where T is the clock period driving the switch in Figure 6b, *V_out_(ω)* is a low-frequency signal, and the last term in Equation (16) can be approximated as 0. Assuming that *V_in_(ω)* is also a low-frequency signal and that *|ω| > ω*_0_/2, *V_in_(ω−kω*_0_*)* can be ignored as well. Since g_0_ = *D*/*R*, Equation (16) can be simplified to

(17) $$\frac{V_{out}}{V_{in}}(j\omega )=\frac{1}{1+j\omega C\frac{R}{D}}$$

In the above case, the resistor *R* amplifies 1/*D* times. With a clock pulse width of 1 ns and a clock frequency of 5 kHz, a duty cycle of 1/200,000 can be realized, and a 1 MΩ resistor can be equivalently enlarged to 200 GΩ. Designing the integrator feedback capacitance *C*_int_= 20 pF, a high pass cutoff frequency of 0.16 Hz can be realized for the instrumentation amplifier.

### 3.3. Design of Ripple Rejection Loop (RRL)

Input offset voltage due to device mismatch is modulated to high frequencies by the chopper, and the chopper ripple of the CCIA can easily saturate the amplifier, making it unable to amplify the bio-signal to its design value. Due to the limited bandwidth of the op-amp, it will present a high-frequency ripple voltage at the output [21], which has an amplitude of

(18) $$V_{ripple}=\frac{V_{OS}\cdot G_{m1}}{2\cdot f_{chop}\cdot C_{m1,2}}$$

where *V_OS_* is the input offset voltage of the main op-amp, *G_m1_* is the transconductance of the first stage of the transconductance amplifier of the main op-amp, and *C_m1,2_* is the Miller compensation capacitance of the main op-amp. Assuming that the main op-amp’s offset voltage *V_OS_* is 5 mV, *G_m1_* is 10 μS, and the compensation capacitance is 5 pF, the output ripple amplitude under the chopping frequency of 5 kHz is 250 mV. Such a large output ripple will cause serious interference with the output signal.

**Figure 7:**
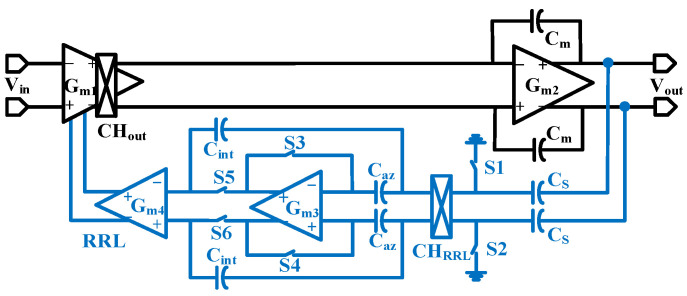
The structure of the ripple rejection loop.

A negative feedback loop RRL is designed to suppress the output high-frequency ripple; its circuit structure is shown in Figure 7. The RRL mainly consists of a sampling capacitor C_S_, a chopper switch CH_RRL_, an integrator composed of a transconductance amplifier G_m3_, and a transconductance amplifier G_m4_. The high-frequency ripple at the output of the op-amp is converted to current by capacitor C_S_ and then demodulated to low-frequency ripple current by the chopper switch, and the integrator composed of G_m3_ continuously integrates the demodulated low-frequency ripple and is finally converted to current by transconductance amplifier G_m4_, to be fed back to the input stage of the first-stage transconductance amplifier of the main op-amp, which is offset with the current generated by the op-amp detuned voltage at the input stage of the main op-amp to realize the elimination of the ripple.

**Figure 8:**
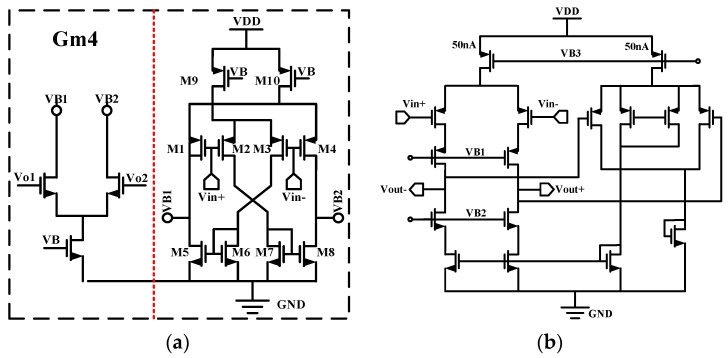
Schematics of (**a**) G_m4_ and (**b**) G_m3_.

Figure 8a gives a diagram of the G_m4_ structure and the feedback nodes of the ripple rejection loop. In the ripple rejection loop, the integrator utilizes a self-zeroing structure to avoid new ripples from the G_m_ input offset voltage. Its operation is divided into two phases: the self-zeroing phase and the integrator phase. 

In the Φ1 stage, switches S1~S4 are disconnected, and S5 and S6 are closed, which realizes the integration of low-frequency ripples and stores them on the capacitor C_int_. In the Φ2 stage, switches S1~S4 are closed, and S5 and S6 are disconnected. In this stage, the transconductance amplifier G_m3_ is in the unit gain negative feedback loop, which stores the out-of-phase voltage of G_m3_ on the capacitor C_az_ and, at the same time, realizes that the integration voltage on C_int_ maintains the integration result of the Φ1 stage. This enables the elimination of high-frequency ripples by generating the required compensation current in both stages. To ensure that both positive and negative cycles of the chopper are used to suppress the ripple, the integrator frequency *f_RRL_* equals 1/2 chopper frequency *f_chop_*. To avoid the ripple rejection loop affecting the effective signal, it is necessary to ensure that *f_RRL_* is much smaller than the chopper frequency *f_chop_*. The ripple rejection loop suppression coefficient is given by the following:

(19) $$L(0)=\frac{g_{m4}\cdot A_{gm3}}{C_{m}\cdot f_{chop}}$$

*A_gm3_* represents the DC gain of the transconductance amplifier G_m3_. To avoid the impact of high-frequency ripple on the output signal, the suppression of the transmission ripple needs to reach at least 40 dB. Therefore, G_m3_ is designed as a single-stage telescopic structure for the input of PMOS transistors, as shown in Figure 8b, with a total current consumption of 100 nA. At such a low current, the DC gain of the single-stage telescopic amplifier is also relatively large enough to meet the demand for ripple suppression with low power consumption.

## 4. Analysis and Design of the Proposed PGA-LPF

To amplify different biological signals to the appropriate amplitude for quantization by the analog-to-digital converter (ADC), a programmable amplifier needs to be introduced into the analog front-end circuit to amplify the biological signals again. To avoid the noise outside the signal band and the high-frequency ripple of the chopper modulation, the circuit needs to introduce a low-pass filter [16]. In addition, for different biological signals with different frequency bands, it is necessary to realize that the bandwidth of the low-pass filter can be adjusted. Due to the low-frequency characteristics of biological signals, the main design difficulty is how to realize an ultra-low cutoff frequency in a small area while taking into account the design requirements of low power consumption. 

**Figure 9:**
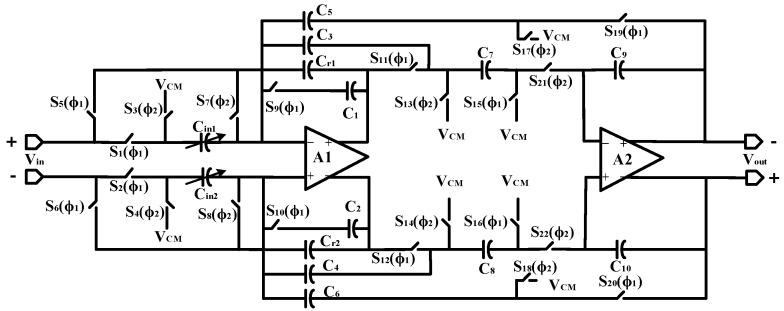
Schematic of used PGA-LPF.

The schematic of the PGA-LPF circuit used in this paper is shown in Figure 9. It is mainly composed of operational amplifiers A1 and A2, switch S_1_~S_22_, capacitors C_1_~C_10_, and input capacitors C_in1,2_. The switches are controlled by a non-interleaved clock. A capacitive feedback network is added to the capacitive low-pass filter, and the gain is adjustable by adjusting the capacitance, thus constituting a combination circuit of PGA and LPF. The structure uses a fully differential structure to improve the overall common mode rejection ratio of the circuit. 

The circuit transfer function is

(20) $$H(s)=\frac{H_{0}\omega_{0}}{s^{2}+\frac{\omega_{0}}{Q}+\omega_{0}^{2}}=\frac{f_{s}^{2}\frac{C_{in1,2}C_{7,8}}{C_{1,2}C_{9,10}}}{s^{2}+f_{s}\frac{C_{3,4}}{C_{1,2}}+f_{s}^{2}\frac{C_{5,6}C_{7,8}}{C_{1,2}C_{9,10}}}$$

Therefore, the passband gain of the PGA-LPF is

(21) $$H_{0}=\frac{C_{in1,2}}{C_{5,6}}$$

Equation(21) shows that the DC gain of the switched-capacitor filter is jointly determined by C_in1,2_ and C_5,6_; thus, the DC gain can be changed by adjusting the value of C_in1,2_, realizing the function of programmable gain. The cutoff frequency of the PGA-LPF module is represented as follows:

(22) $$\omega_{0}=f_{s}\sqrt{\frac{C_{5,6}C_{7,8}}{C_{1,2}C_{9,10}}}$$

where *f_s_* is the filter sampling frequency.To collect signals for each phase of the chopping frequency, the sampling frequency is set to twice the chopping frequency. The bandwidth of the switched-capacitor filter can be adjusted by reasonably setting the size of the sampling frequency *f_s_*. The quality factor *Q* is

(23) $$Q=\frac{C_{1,2}}{C_{3,4}}\sqrt{\frac{C_{5,6}C_{7,8}}{C_{1,2}C_{9,10}}}$$

Therefore, the bandwidth and gain of the PGA-LPF module can be adjusted by appropriately controlling the capacitor size and sampling frequency in the circuit. In this paper, the capacitance *C_5,6,7,8_* = 200 fF, *C_1,2,9,10_* = 1.2 pF, and the circuit’s cutoff frequency is 265 Hz. C_in_ consists of three switches and capacitance, which can be constituted by the capacitance values 0.2 pF to 0.8 pF and adjustable step 0.1 pF to realize the gain of 1 to 4 times (step is 0.5). 

**Figure 10:**
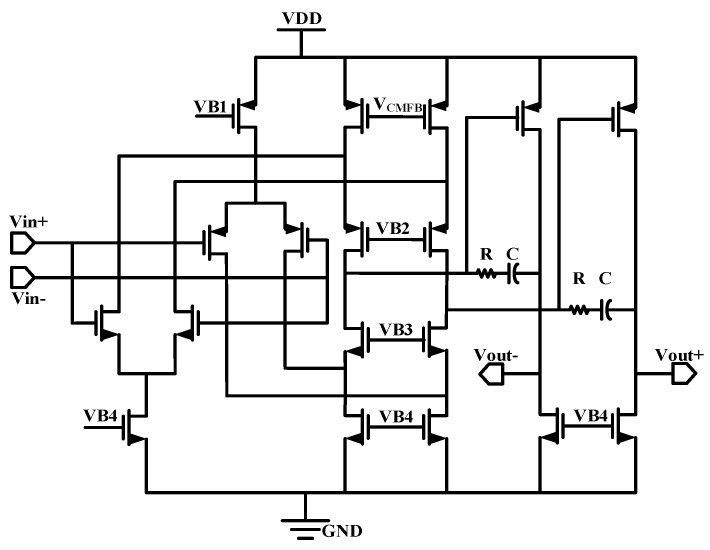
Schematic of the A1 employed in PGALPF.

Since the process of charge transfer, as well as sharing in switched-capacitor circuits, is affected by the gain and the bandwidth of the op-amp, the op-amp needs to have a sufficiently high gain to avoid affecting the circuit’s accuracy. As shown in Figure 10, the core amplifier in the PGA-LPF module uses a fully differential two-stage transconductance amplifier with rail-to-rail input. The first stage of the amplifier utilizes a P-NMOS complementary structure to increase the circuit input swing and a cascode structure to increase the circuit gain. For the second stage, a common source stage circuit is used to increase the output swing of the amplifier.

**Figure 11:**
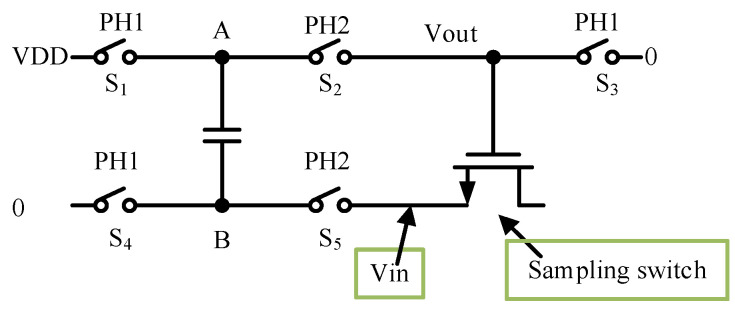
Diagram of the gatevoltage bootstrap switch.

The noise of the PGA-LPF conversion to the input needs to be divided by the closed-loop gain of the instrumentation amplifier; thus, the noise requirement can be relaxed to obtain a lower power consumption of the circuit. A Miller compensation capacitor and a zeroing resistor are added to the circuit to improve circuit stability, and a common-mode feedback circuit is introduced to stabilize the common-mode operating point of the circuit and to improve the circuit’s immunity to common-mode interference. In a switched-capacitor circuit, the switch’s non-ideal factor seriously impacts the acquired signal quality [6]. To improve the linearity of the PGA-LPF circuit, a gatevoltage bootstrap switch is designed as a sampling switch for the PGA-LPF module. The diagram of the gatevoltage bootstrap switch is shown in Figure 11. In the PH1 phase, S_1,3,4_ is closed, and S_2,5_ is disconnected, *V_A_ = V_DD_*, *V_B_ = 0*, *V_out_ = 0*. The charge on the capacitor is

(24) $$Q_{1}=V_{DD}\cdot C+(0-V_{in})C_{g}$$

where *C*_g_ represents the parasitic capacitance of the MOS transistor. In the PH2 phase, switch S_2,5_ is closed, and S_1,3,4_ is disconnected, *V_A_ = V_out_*, *V_B_ = V_in_*. The charge on the capacitor is

(25) $$Q_{1}=(V_{out}-V_{in})\cdot (C+C_{g})$$

According to the conservation of charge, *Q*_1_ = *Q*_2_.

(26) $$\begin{array}{l} V_{DD}\cdot C+(0-V_{in})C_{g}=(V_{out}-V_{in})\cdot (C+C_{g})\\ \Rightarrow V_{out}=\frac{(V_{DD}+V_{in})\cdot C}{C+C_{g}}\approx V_{DD}+V_{in} \end{array}$$

**Figure 12:**
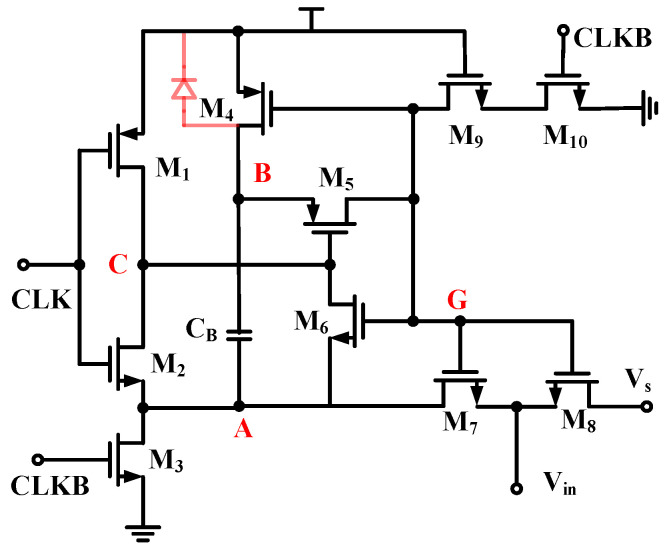
Schematic of the gatevoltage bootstrap switch.

The gate and source voltage difference of the MOS transistor at stage PH1 is a fixed value, and the on-resistance does not change with the input signal; thus, the linearity of the switch can be greatly improved. The schematic of the gatevoltage bootstrap switch is shown in Figure 12. For the convenience of analysis, the key nodes are marked with red letters A, B, C and G. The sampling switch operates in two stages: at stage PH1, CLK is low, CLKB is high, the voltage at both ends of capacitor C_B_ is reset, the voltage at point A is pulled down to 0, the voltage at point B is pulled up to *V_DD_*, the gate of the M_8_ is reset to 0, the voltage at point C is *V_DD_*, and the M_5_ is in the off state; at stage PH2, CLK is high, CLKB is low, the M_9_ and M_10_ discharge Pass off, M_5_ is on, point B and point G charge sharing, and M_7_ is on. According to the conservation of charge at both ends of the C_B_, V_B_ is given by the following expression:

(27) $$V_{B}=V_{G}=V_{DD}+V_{in}$$

The M_8_ in the sampling stage keeps the gate–source voltage difference constant while the gate voltage varies with the input signal. Due to the parasitic capacitance C_g_ in M_8_, the on-state impedance cannot be completely independent of the input signal; the design ensures that C_g_ is much smaller than C_B_. In addition, the voltage of point B can reach a maximum of 2*V_DD_* (*V_in_ = V_DD_*), If the transistor M4 substrate is connected to *V_DD_* via the usual method, there will be a forward diode between point B and *V_DD_*, which results in a maximum point B voltage of only *0.7 V + V_DD_*. The transistor M_4_ substrate needs to be shorted with point B to avoid voltage clamping. 

## 5. Results and Discussion

**Figure 13:**
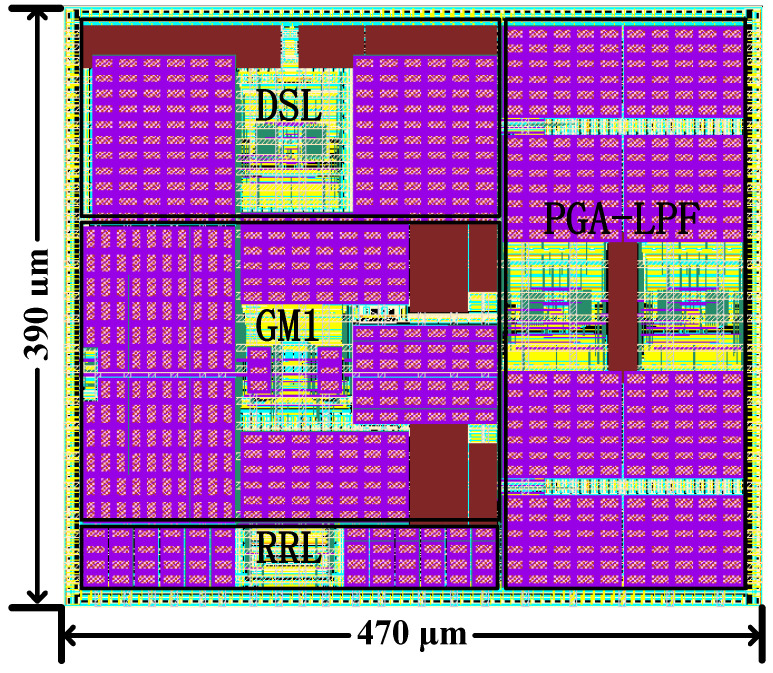
The overall layout of the proposed AFE.

Figure 13 shows the layout design of the proposed AFE, which includes GM1, DSL, RRL and PGA-LPF. It is implemented using the UMC 40 nm CMOS process with an area of 0.18 mm2. The power consumption after the layout simulation is 8.94 μW at 1.2 V supply. 

**Figure 14:**
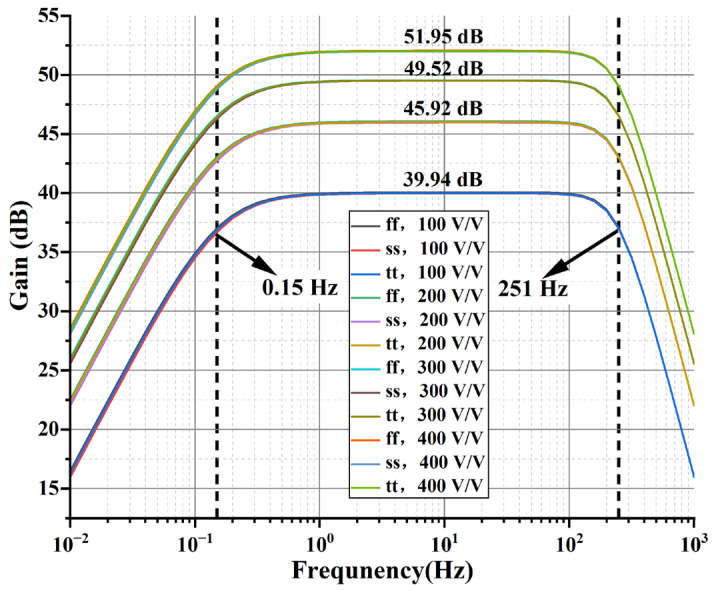
The amplitude and frequency responses of the proposed AFE.

The overall AFE amplitude–frequency characteristic simulation results are shown in Figure 14. The switching configuration through the PGA-LPF module enables the 100 *V*/*V*–400 *V*/*V* gain to be adjustable. The gains in the mid-frequency bandwidth are 39.94 dB, 45.92 dB, 49.52 dB, and 51.95 dB, respectively. The high-pass characteristics are determined by the DSL loop with a high-pass cutoff frequency of 0.15 Hz, and the low-pass characteristics are determined by the PGA-LPF module with a low-pass cutoff frequency of 251 Hz.

**Figure 15:**
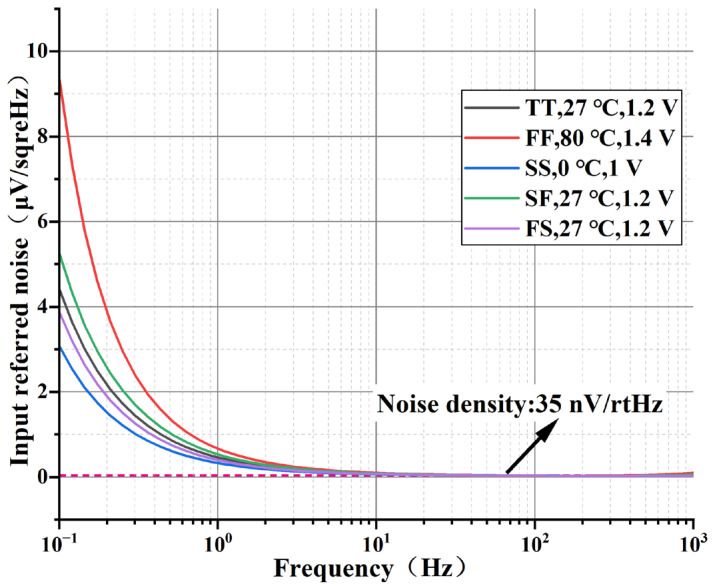
The input referred noise performance of the proposed AFE.

The equivalent input noise of the analog front-end circuit under different Process Voltage Temperature (PVT) conditions is given in Figure 15. Comparing the system noise under different process angles, the circuit equivalent input noise curve does not change much over the frequency range of the signal of interest. The input noise is integrated across the frequency interval ranging from 0.5 Hz to 1 kHz. The results of the integrated noise are presented in Table 1 andare approximately 1 μVrms. The simulation results show that, with the chopping frequency employed and the corner frequency depicted in Figure 15, the chopper stabilization suppresses 1/f noise.

**Figure 16:**
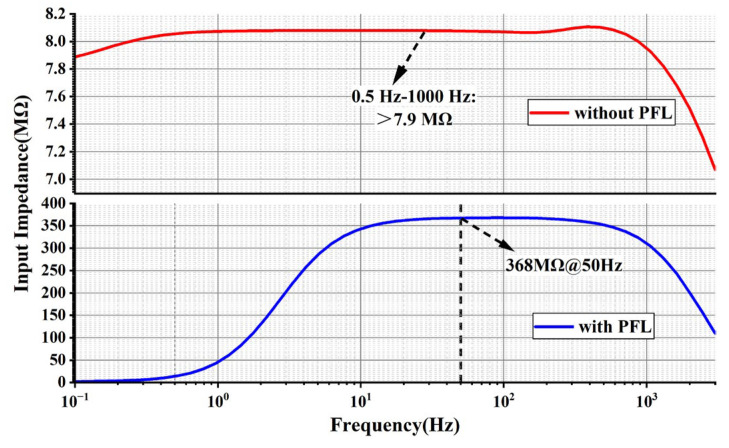
The input impedance comparison of AFE (**top**) without PFL and (**bottom**) with PFL.

As is shown in Figure 16., without the introduction of positive feedback capacitance, the input impedance of the circuit is about 7.9 MΩ in the signal bandwidth. After the introduction of a positive feedback capacitor loop, the input impedance of the circuit is more than 300 MΩ at 20~500 Hz. The input impedance at 50 Hz can reach up to 368 MΩ, which is small compared with the theoretical design value due to the parasitic capacitance of the op-amp itself; the chopper switch also constitutes an AC resistor. 

**Figure 17:**
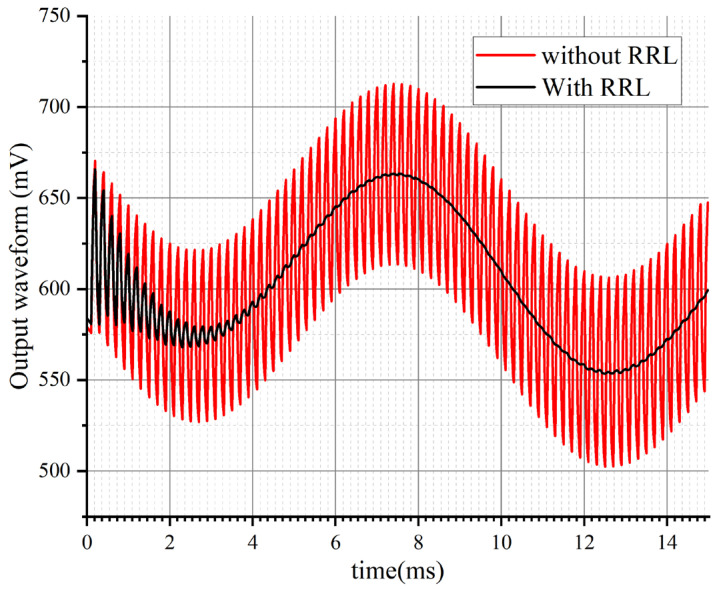
The amplitude and frequency response of PGA-LPF with different bandwidths.

Simulation verification was conducted on the RRL function by adding a 1 mV offset voltage at the output end of the main amplifier for transient simulation. Figure 17 shows the output transient waveforms with and without the RRL. From the figure, it can be seen that the RRL has a significant effect on the elimination of output ripple. After about 5 ms of stabilization, the ripple is suppressed and will not affect the output signal. The residual high-frequency ripple will be eliminated a second time after passing through the next stage of filtering.

**Figure 18:**
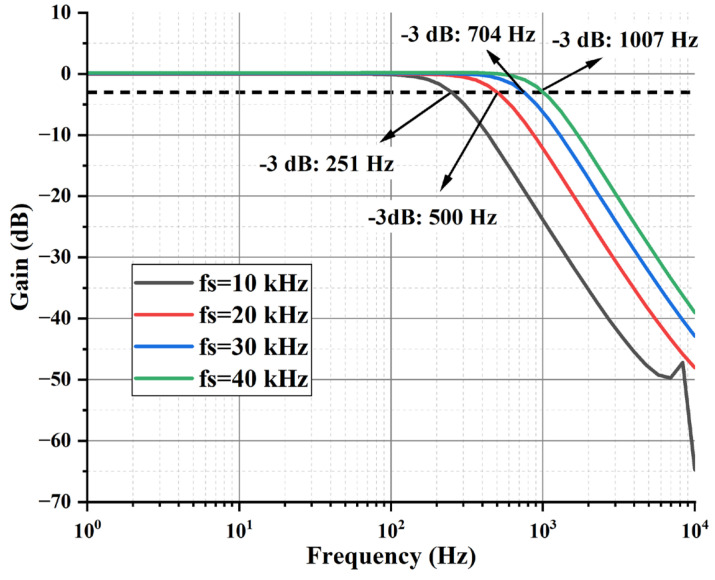
The amplitude and frequency response of PGA-LPF with different bandwidths.

The amplitude and frequency response of the PGA-LPF module is simulated in Figure 18. When the sampling frequency fs is taken as 10 kHz, 20 kHz, 30 kHz, and 40 kHz, it can be seen that the bandwidth of the PGA-LPF module is about 251 Hz, 500 Hz, 704 Hz, and 1007 Hz, respectively. This is slightly different from the design value due to the effect of parasitic capacitance.

**Figure 19:**
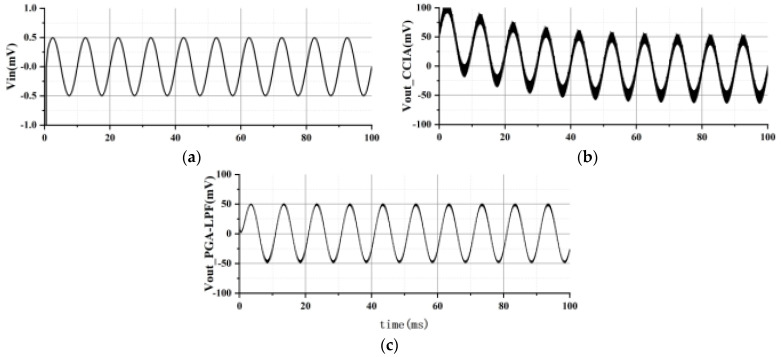
The transient response of proposed AFE with (**a**) input signal, (**b**) CCIA output signal, and (**c**) PGA-LPF output signal.

A differential signal with a frequency of 100 Hz and an amplitude of 1 mVpp is input to simulate the EEG signal, and the PGA–LPF gain is set to 1 x for transient simulation. The simulation results are shown in Figure 19. It can be seen that CCIA achieves 40 dB amplification of the signal. The PGA-LPF filters the signal to filter out the high-frequency ripples and realize normal amplification of differential signals, which is consistent with the expectation. A comparison of theperformance with other related works is shown in Table 2. Compared with other reporteddesigns, the design presented in this work achieves good noise and CMRR when implemented in the UMC 40 nm CMOS process.

## 6. Conclusions

This paper describes a 35 nV/√Hz analog front-end circuit with bandwidth-gain adjustable using a 40 nm CMOS process. The combination of the chopping technique and transconductance-boosting technique is used to greatly reduce the 1/f noise, and DSL and RRL are designed for the elimination of the DC offset and suppression of high-frequency ripples, respectively. Moreover, The PGA-LPF circuit is introduced to realize the bandwidth-gain adjustable. The post-simulation results show that the AFE has an integrated equivalent input noise of 1.04 μVrms, a 50 mV electrode offset voltage cancellation, a common-mode rejection ratio of 111 dB, and 40~52 dB gain adjustability and 250~1 kHz bandwidth adjustability, which can meet the requirements of biopotential signal acquisition.

## Figures and Tables

**Table 1:** Integral noise with different PVT (0.5 Hz~1 kHz).

PVT	TT, 1.2 V 27 °C	SS, 1 V 0 °C	FF, 1.4 V 80 °C	FS, 1.2 V 27 °C	SF, 1.2 V 27 °C
Integral noise (μVrms)	1.03	0.71	2.26	0.83	1.41

**Table 2:** Performance comparison with other related works.

Reference	[21]	[17]	[22]	[23]	[24]	[25]	This work
Process (nm)	180	65	40	180	180	180	40
Supply (V)	1	1	1.2	1.2	1.2	1.8	1.2
Area (mm^2^)	0.19	0.1	0.069	0.062	N/A	0.072	0.18
Power (μW)	2.14 ^a^	1.8 ^a^	2.8 ^a^	0.507 ^a^	0.8 ^a^	3.66 ^a^	8.94 ^b^
IRN (μVrms)	2.1 (1–200 Hz)	6.7 (0.5–100 Hz)	7.1 (1–5 kHz)	0.67 (0.5–150 Hz)	1.7 (0.5–150 Hz)	2.86 (1–5 kHz)	1.0360 (0.5–1 kHz)
Noise density (nV/√Hz)	133	60	80	85	N/A	N/A	35
Zin (Ω)	4.46 G @0.01 Hz	30 M @10 Hz	1.6 G	150 M @1 Hz	N/A	1.8 G @10 Hz	368 M @50 Hz
Bandwidth (Hz)	0.5–750	0.5–100	0.5–5 k	0.1–1.2 k	1–900	0.1–5.1 k	0.5–1 k
Gain (dB)	40	40	25.7	30–45	43–57	26.04	40–52
CMRR (dB)	N/A	>134	>78	>65	85	N/A	>111
Results	Measure	Measure	Measure	Simulation	Simulation	Simulation	Simulation

a: the power of IA, b: the power of CCIA and PGA-LPF.

## Data Availability

Data are contained within the article.
